# ROS generating BODIPY loaded nanoparticles for photodynamic eradication of biofilms

**DOI:** 10.3389/fmicb.2023.1274715

**Published:** 2023-10-12

**Authors:** Charlotte Kromer, Karin Schwibbert, Sebastian Radunz, Dorothea Thiele, Peter Laux, Andreas Luch, Harald R. Tschiche

**Affiliations:** ^1^Department Chemicals and Product Safety, Product Materials and Nanotechnology, German Federal Institute for Risk Assessment, Berlin, Germany; ^2^Institute of Pharmacy, Freie Universität Berlin, Berlin, Germany; ^3^Department Materials and the Environment, Biodeterioration and Reference Organisms, Federal Institute for Materials Research and Testing, Berlin, Germany; ^4^nanoPET Pharma GmbH, Berlin, Germany

**Keywords:** biofilm, disinfection, antimicrobials, photodynamic therapy, aPDT, nanoparticles, BODIPY

## Abstract

Bacterial biofilms can pose a serious health risk to humans and are less susceptible to antibiotics and disinfection than planktonic bacteria. Here, a novel method for biofilm eradication based on antimicrobial photodynamic therapy utilizing a nanoparticle in conjunction with a BODIPY derivative as photosensitizer was developed. Reactive oxygen species are generated upon illumination with visible light and lead to a strong, controllable and persistent eradication of both planktonic bacteria and biofilms. One of the biggest challenges in biofilm eradication is the penetration of the antimicrobial agent into the biofilm and its matrix. A biocompatible hydrophilic nanoparticle was utilized as a delivery system for the hydrophobic BODIPY dye and enabled its accumulation within the biofilm. This key feature of delivering the antimicrobial agent to the site of action where it is activated resulted in effective eradication of all tested biofilms. Here, 3 bacterial species that commonly form clinically relevant pathogenic biofilms were selected: *Escherichia coli*, *Staphylococcus aureus* and *Streptococcus mutans*. The development of this antimicrobial photodynamic therapy tool for biofilm eradication takes a promising step towards new methods for the much needed treatment of pathogenic biofilms.

## 1. Introduction

Biofilms are microbial communities adhered to surfaces and surrounded by a self-produced matrix, mainly composed of water and extracellular polymeric substances (EPS) (Flemming and Wingender, 2010). The matrix plays an important role in biofilm formation by providing the biofilm with structural integrity as well as increased resistance to external influences such as temperature changes, desiccation, shear forces and also disinfection (Donlan and Costerton, 2002; Flemming and Wingender, 2010). In addition, it forms a reservoir for nutrients and allows the microorganisms to establish long-term synergistic associations characterized by social interactions and community evolution (Hansen et al., 2007; Flemming et al., 2016).

Infections caused by bacteria including those involving biofilms pose a global threat to human health. Particularly devastating is the ever-evolving resistance of bacteria to existing treatments. Biofilm formation protects bacteria from common cleaning procedures like disinfection and allows further colonization (Dang and Lovell, 2016). Strategies for biofilm control currently used include cleaning and disinfection, material selection and surface treatments such as application of ultraviolet light, plasma, and ultrasonic treatment. In the medical field and for application in humans, the use of antibiotics plays a fundamental role. Most of these methods are primarily aimed at killing planktonic bacteria or inhibiting their growth and are, therefore, not sufficient to control biofilms entirely (Ciofu et al., 2022). Two problems are recognized to be inextricably linked to this approach: (I) the frequently observed development of resistance to antimicrobial agents and (II) the fact that therapeutic agents are much less effective on bacteria growing in biofilms compared to planktonic cells (Mah and O'Toole, 2001). The latter point is of particular importance because in recent years there has been mounting evidence that most chronic bacterial infections are associated with biofilms (Macia et al., 2014; Kolpen et al., 2022). The rapid development of resistance in many bacterial species is also highly problematic as it makes future eradication even more challenging. Therefore, strategies to overcome bacterial persistence by inhibiting biofilm formation or removing mature biofilms that are effective and can be used long term are urgently needed.

A great hope lies in the utilization of *in situ* generated reactive oxygen species (ROS). This concept is already applied in photodynamic therapy (PDT). PDT uses photosensitizers (PS) that are activated by visible or near-infrared light but are non-toxic without illumination. The PS first forms an excited singlet state, followed by a transition to the long-lived excited triplet state, which undergoes photochemical reactions in the presence of oxygen and generates ROS (Bekmukhametova et al., 2020). The ROS can then destroy biological targets such as cancer cells and microbes including bacteria (Hu et al., 2018). When PDT is applied to combat microbes, such as bacteria, it is often referred to as antimicrobial photodynamic therapy (aPDT). A key advantage of aPDT is that ROS damage is completely non-specific and, thus, can be used on all bacteria, as there is no known resistance to ROS (Hughes and Webber, 2017). Commonly used classes of PS are porphyrins, squarains, phenothiazines and boron-dipyrromethene (BODIPY) (Bekmukhametova et al., 2020). BODIPY dyes are excellent candidates for the development of modern aPDT strategies due to their particularly remarkable photophysical properties, such as high molar absorption coefficients, high quantum yields for ROS generation and high photostability (Rebeca and Jorge, 2018; Boens et al., 2019; Radunz et al., 2020). Additionally, they are a highly versatile dye class that can be easily prepared and structurally altered to benefit various applications (Bañuelos, 2016; Sheng et al., 2019; Radunz et al., 2020).

Generally, the applicability of many PS molecules is limited by their poor water solubility, aggregation behavior and impaired ability to sufficiently penetrate tissues and biofilms (Abrahamse and Hamblin, 2016; Songca and Adjei, 2022). In addition, the lifetime of the generated singlet oxygen is very short, limiting its diffusion to only 10–55 nm (Dysart and Patterson, 2005). Therefore, the photodynamic damage is likely to occur only in close proximity to the location of the PS, thus, the ROS generation has to be induced at the target site, e.g., inside the biofilm (Moan et al., n.d.). The higher the concentration of the PS inside the biofilm, the better the therapeutic performance (Bekmukhametova et al., 2020).

Nanoparticles (NPs) can be utilized to act as carriers for the PS to facilitate the delivery and accumulation within the biofilm. By using hydrophilic particles in which the dye is embedded, the solubility issues of the mostly hydrophobic PS can be circumvented. In addition, there is the possibility of using NPs that can enable active targeting, e.g., by binding to specific cell components or biofilm structures, e.g., by utilizing protein conjugation. However, the NPs accumulation in the biofilm is also possible in a passive manner through diffusion, influenced by their charge, size or hydrophobicity (De-la-Pinta et al., 2019; Hollmann et al., 2021). This allows high amounts of PS to be concentrated locally at the target site, especially if the particles have a high dye loading.

Several studies have shown the bactericidal effect of aPDT utilizing NPs, e.g., methylen blue-loaded gold NPs (Khan et al., 2017) and chitosan NPs (Yaghoubi et al., 2020), curcumin-loaded PLGA NPs (Raschpichler et al., 2019) toluidine blue-loaded alginate NPs (Usacheva et al., 2016) and rose bengal-loaded chitosan NPs (Shrestha et al., 2014). However, there are limitations to the published methods that constrain their successful use for biofilm removal. For example, elimination rates lower than 2 log units achieved with toluidine blue or methylen blue-loaded NPs cannot be viewed as sufficient biofilm removal (Klepac-Ceraj et al., 2011; Usacheva et al., 2016; Anju et al., 2019). This is particularly important as biofilms can proliferate and rebuild their population quickly, even if only a few bacterial cells survive. The commonly used PS Rose Bengal was also used in several NPs applications but showed relevant dark toxicity interfering with the aPDT treatment (Shrestha et al., 2014; Anju et al., 2018). The use of highly effective yet controllable PS for photodynamic eradication of biofilms is therefore urgently needed.

In general, the effect of aPDT on planktonic bacterial cells is higher than on biofilms. Most published methods are able to eradicate planktonic bacteria by several log units; however, for biofilms, the reduction is only in the double-digit percentage range (Anju et al., 2018; Dai et al., 2018; Anju et al., 2019). Reasons for this are the more difficult penetration of PS into biofilms due to EPS and the generally higher concentration of bacteria in the biofilm compared to a suspension in medium (Li et al., 2023). In addition, bacteria in different physiological stages are present in a biofilm, which means that not all of them are in a vulnerable, proliferating state (Ciofu et al., 2022). Some may be in a dormant state with a lower metabolism, which also limits the internalization of substances and thus the effectiveness of many treatment agents. It is therefore important to develop systems that are also highly effective in biofilms.

It is well-known, that gram-positive bacteria are more susceptible to aPDT than gram-negative bacteria, due to their cell wall composition (Bekmukhametova et al., 2020). Studies have confirmed this fact and shown that gram-negative bacteria are up to 10 times less sensitive than gram-positive bacteria (Yang et al., 2012; Kirar et al., 2018). Therefore, investigations for utilizing aPDT for biofilm eradication should also be carried out on gram-negative as well as gram-positive bacteria. On top, the functionality and also the limitations of aPDT in relation to the composition of the cell wall should be investigated further.

The efficacy of aPDT systems can be significantly increased by the development of more complex and sophisticated aPDT systems, for example in combination with other antibacterial agents (Pérez-Laguna et al., 2019; Songca and Adjei, 2022). However, when considering these developments in terms of their practicability, it is at least as important to develop systems that are easy to manufacture and use and yet highly effective.

In this study, a highly effective PS combined with a facile NP delivery system was utilized to overcome the limitations of previously described aPDT systems to achieve effective eradication of both, planktonic bacteria and biofilms. For this purpose, a highly effective diiodinated BODIPY derivative that can be excited with visible light with a wavelength of 530 nm was embedded in polystyrene NPs. Both Gram-positive and Gram-negative biofilm-forming bacteria with high clinical relevance were selected to evaluate the effectiveness of the BODIPY-loaded NPs for aPDT of planktonic bacteria and biofilms.

## 2. Methods

### 2.1. Materials and reagents

All solvents (tetrahydrofuran (THF), ethanol, acetonitrile and dimethylsulfoxide) were of analytical grade, purchased from Merck and Thermo Fisher Scientific, and used as received. The 100 nm polystyrene NPs were purchased from Kisker Biotech and ultrasonicated prior to use. *Escherichia coli* (*E. coli*)*, Staphylococcus aureus* (*S. aureus*), and *Streptococcus mutans* (*S. mutans*) were purchased from DSMZ-German collection of microorganisms and cell cultures. All cell culture materials and ingredients were obtained from Merck, VWR, and Thermo Fisher Scientific.

### 2.2. BODIPY synthesis

The synthesis as well as analytical and optical characterization of the iodinated BODIPY dye has been reported previously (Radunz et al., 2020). For this study, 4,4-difluoro-1,3,5,7-tetramethyl-8-(4-hydroxyphenyl)-bora-3a, 4a-diaza-s-indacene was used as precursor dye for the synthesis of the diiodinated singlet oxygen-generating BODIPY (Radunz et al., 2017). All compounds subjected to biological assays were of >95% purity (ultra performance liquid chromatography). All reagents and solvents employed for the synthesis and characterization were used without further purification.

N-Iodosuccinimide (2.2 equiv.) was added slowly in small portions to a stirred solution of the precursor BODIPY (1 equiv.) in 100 mL of dichloromethane. After complete addition of the N-Iodosuccinimide, the reaction mixture was stirred for further 60 min. Then, the reaction mixture was washed with deionized water and subsequently dried over MgSO_4_. Purification was performed by column chromatography in the dark using dichloromethane/petroleum ether (1/1, v/v) as eluents followed by recrystallization by chloroform/n-hexane.

4,4-Difluoro-2,6-diiodo-1,3,5,7-tetramethyl-8-(4-hydroxyphenyl)-bora-3a,4a-diaza-s-indacene. Yield 23%; UPLC: >95% purity; 1H NMR (500 MHz, CDCl3) [ppm]: *δ* = 7.04 (d, 2H_aryl_), 6.98 (d, 2H_aryl_), 2.63 (s, 6H_methyl_), 1.48 (s, 6H_methyl_); 13C NMR (125 MHz, CDCl3) [ppm]: *δ* = 158.2, 156.3, 145.5, 142.2, 131.8, 129.0, 125.4, 116.3, 85.3, 17.0, 15.8; MS (ESI-TOF): m/z calculated for C19H17BF2I2N2NaO^+^ [M + Na]+: 614.9389; found: 614.9417.

### 2.3. Nanoparticle preparation

The nanosensor was prepared from commercially available polystyrene NPs and a diiodinated BODIPY derivative. The BODIPY dye was incorporated into the NPs via a swelling procedure published by Behnke et al. (2011). In brief, BODIPY was first dissolved in THF in a concentration of 3 nmol/1 μL. Dye loading of the NPs was performed by addition of 100 μL of the BODIPY-containing solution to 600 μL of an aqueous suspension of the NPs (5 mg/mL). The suspension was shaken for 30 min at room temperature, followed by 40 min centrifugation at 16,000 g (Eppendorf centrifuge 5415D). The supernatant consisting of unembedded BODIPY dye was removed from the BODIPY-loaded NPs. The purification steps consisted of a total of 5 washing steps starting with MilliQ water, followed by 2 washing steps with ethanol and 2 final washing steps with MilliQ water.

### 2.4. Particle size and zeta potential

The particle size (hydrodynamic diameter) and polydispersity index (PDI) of the NPs was determined by dynamic light scattering (DLS) (Zetasizer Malvern Panalytical, Malvern Nano ZS). Measurements were carried out in MilliQ water at 50 μg/mL in a quartz glass cuvette. Thermal equilibration time was set to 60 s at 25°C. Each intensity-weighted size distribution represents the average of 10 individual DLS analyses and three independent replicates. A Dip cell kit (Malvern Panalytical) was used for the determination of the zeta potential. The average of 10 individual zeta potential analyses and three independent replicates were determined. The particle size was also assessed using a transmission electron microscope (TEM). Formvar coated copper and gold grids with 400 mesh and 3.5 mm diameter (Plano GmbH, Germany) were hydrophilized with 0.2% alcian blue (Sigma Aldrich, Germany) in 0.03% acetic acid solution. The grids were floated on alcian blue droplets for 10 min, and dried using a filter paper. 5 μL of a 1 mg/mL NPs dispersion was applied immediately on each hydrophilized grid, incubated for 1 min and the excess liquid was removed with a filter paper. Samples on the copper and gold grids were observed in a Jeol 1,400 Plus TEM (Jeol GmbH, Germany) operated at 120 kV. Material identification was done using diffraction pattern from published resources. Imaging was performed using a Veleta G2 camera (Olympus, Germany). Particle size was measured using iTEM software provided by Olympus. At least 4 different areas of each grid were examined per sample.

### 2.5. Dye loading

Dye loading was determined by a spectrophotometric method (FoodALYT, Germany). A calibration curve for the absorbance of BODIPY in THF at 530 nm was prepared to evaluate the dye loading. The concentration range of 2.5–20 nmol/mL was linear with *R* = 0.9813 determined by a linear regression model. As a second step, three different volumes of BODIPY-loaded NPs suspension were added to Eppendorf tubes and centrifuged at 16.000 g for 40 min. The supernatant was removed, and the NPs were dissolved in 1000 μL THF. The absorption at 530 nm was determined in a spectrophotometer. The dye concentration in 1000 μL THF was calculated as dye equivalents to the calibration curve. Lastly, the dye loading per mg NPs was calculated.

### 2.6. ROS assay

Singlet oxygen generation by the BODIPY dye was quantified by an indirect method using 1,3-diphenylisobenzofuran (DPBF) as singlet oxygen quencher (Krieg, 1993; You, 2018). The decrease of absorbance of DPBF at 410 nm upon quenching of singlet oxygen produced by excitation of the BODIPY dye was monitored. Therefore, the absorbance of DPBF was set to values of about 0.8 at the absorption maximum and the absorbance of the respective dye was set to absorbances of 0.1 which was approx. 15 nmoL/mL. Subsequently, the samples were illuminated stepwise using a 530 nm LED array (LEDA-G, Teleopto Bio Research Center Co., Japan). The initial LED light power of 43.5 mW/cm^2^ was adjusted with regard to the sensitivity of the assay molecule and scaled down to around 8.7 mW/cm^2^. After each illumination step, an absorption spectrum was recorded and the rate constants *k* were determined assuming pseudo-first-order kinetics with [DPBF]_0_ being the absorbance (area under the curve) at time point 0 and [DPBF] at the measured time point (Wang et al., 2015; Hu et al., 2018).


$$kt=\ln\frac{{\left[\mathrm{DPBF}\right]}_{0}}{\left[\mathrm{DPBF}\right]\;}$$

### 2.7. Bacterial cell culture and biofilm growth

*Escherichia coli* TG1 DSM 6056, *Staphylococcus aureus* BAM 480 and *Streptococcus mutans* DSM 20523 were used as biofilm forming microorganisms (Schwibbert et al., 2019). *E. coli* and *S. aureus* were cultivated on Luria–Bertani (LB) medium agar plates and passaged every 3–4 weeks. *S. mutans* was cultivated on Columbian blood agar plates and passaged every 1–2 weeks. For all biofilm experiments, 20 mL liquid medium was inoculated with single colonies and cultured overnight at 37°C with shaking at 120 rpm on an orbital shaker (Incubating orbital shaker, Professional 3,500, VWR) 57. LB medium was used for *E. coli*, AB medium for *S. aureus* and M92 medium for *S. mutans*. The cultures were diluted 1:100 in fresh medium and incubated for additional 1–2 h at 37°C until cells reached the exponential growth phase. For biofilm formation, the optical density of the suspension was measured at 600 nm (Novaspec Plus, Amershan Biosciences) and adjusted to 0.01 (corresponding to approx. 1.2 × 10^6^ cells/mL for *E. coli*, 1.5 × 10^6^ cells/mL for *S. aureus* and 6 × 10^5^ cells/mL for *S. mutans*). *E. coli* biofilms were grown in M9 minimal medium, supplemented with 1 mM thiamine and 20 mg/L proline. *S. aureus* biofilms were grown in M9 minimal medium, supplemented with 1 mM thiamine, 20 mg/L proline, 0.5 g/L protein hydrolysate amicase (acid hydrolyzed casein), and 0.5 g/L yeast extract. *S. mutans* biofilms were grown in M92 medium. For the biofilm assay, biofilms were grown in 96 well plates (100 μL per well) on the well/liquid medium interface for 24 h with constant shaking at 100 rpm for *E. coli* and 60 rpm for *S. aureus* and *S. mutans*. For microscopy, biofilms were formed in ibidi glass slides (8 well chamber slide, Ibidi GmbH) on the glass/liquid medium interface.

### 2.8. Biofilm assay and illumination

For the biofilm assay, the supernatant of the biofilms was removed, and the biofilms were washed with PBS before the NPs were added at a final concentration of 1 mg/mL. The well plate containing the samples and control samples without NPs were staged on top of an LED array standing inside a shaking incubator. The excitation wavelength of the LED array (LEDA-G, Teleopto Bio Research Center Co., Japan) was 530 nm. Samples were illuminated at 43.5 mW/cm^2^ for 30 min, 2 h and 4 h with light doses of 81 J/cm^2^, 324 J/cm^2^ and 648 J/cm^2^, respectively. For each illuminated plate, a dark control was conducted with identical samples without illumination. After the illumination, the plates were taken from the shaker and the supernatant was removed from each well. For quantification of bacteria per biofilm, all samples were washed with PBS and the biofilms were resuspended in 100 μL PBS. Crystal violet staining (0.1%, 10 min incubation, 3 × washing with MilliQ water) and microscopy of the empty wells revealed no leftover biofilm in the wells after this procedure. The PBS containing the biofilm bacteria was serially diluted in a new 96 well plate. These serial dilutions were seeded on LB agar plates, incubated at 37°C over night and the Colony forming units (CFU) were counted.

### 2.9. Imaging (CLSM and SEM)

For confocal laser scanning microscopy (CLSM), all biofilms were imaged at 37°C in Ibidi slides using a Leica SP8 X CLSM equipped with a supercontinuum white light laser and a monochromator (Leica Microsystems). A 100 × / (N.A.1.4) objective with oil immersion was used for imaging. XY images were acquired with 2048 × 2048 or 8,192 × 8,192 pixels and Z-stacks in XYZ mode with 512 × 512 pixels, respectively. To obtain the Z-stacks, images were taken at 1 μm spacing through the biofilm.

For scanning electron microscopy (SEM) imaging, biofilms were grown on 1 × 1 cm glass slides, washed three times with PBS and fixed with 2% glutaraldehyde for 2 h. After three additional washing steps with PBS, specimens were dehydrated in a graded alcohol series (30, 50, 70, 90, and 99% ethanol). Critical point drying was performed with liquid carbon dioxide as a transitional fluid (EM CPD300, Leica, Germany). Biofilm specimens were sputter coated with a 30 nm conducting layer of gold (EM ACE600 table-top coater, Leica, Germany) and examined with an emission scanning electron microscope (XL 30 ESEM, FEI, Netherlands) using secondary electron detector and operated at an electron accelerating voltage of 20 kV and 25 kV. A minimum of three random sections per sample were analyzed.

### 2.10. Statistics

Data analysis was performed with Graph Pad Prism 9. Error bars indicate the standard error of the mean of three independent experiments performed in triplicates. *, **, and *** represent the significant difference to the control determined with an unpaired *t* test with *p* < 0.05, *p* < 0.01, and *p* < 0.001, respectively.

## 3. Results

### 3.1. Design and preparation of the NPs

**Figure 1:**
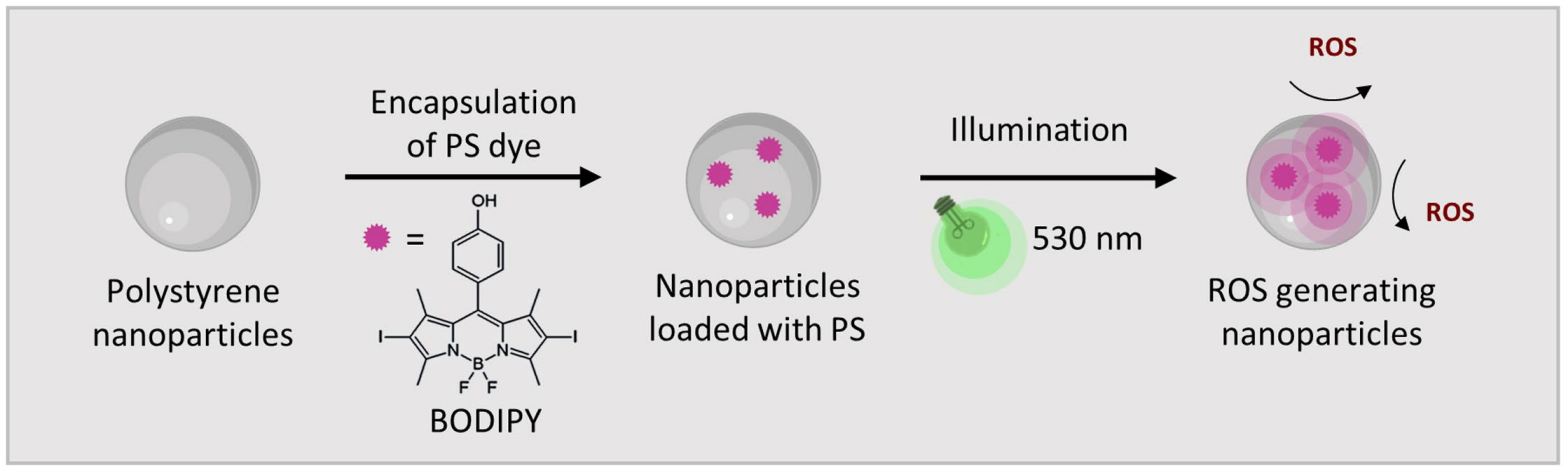
The BODIPY dye embedded in polystyrene NPs generates ROS when activated by visible light (530 nm).

**Figure 2:**
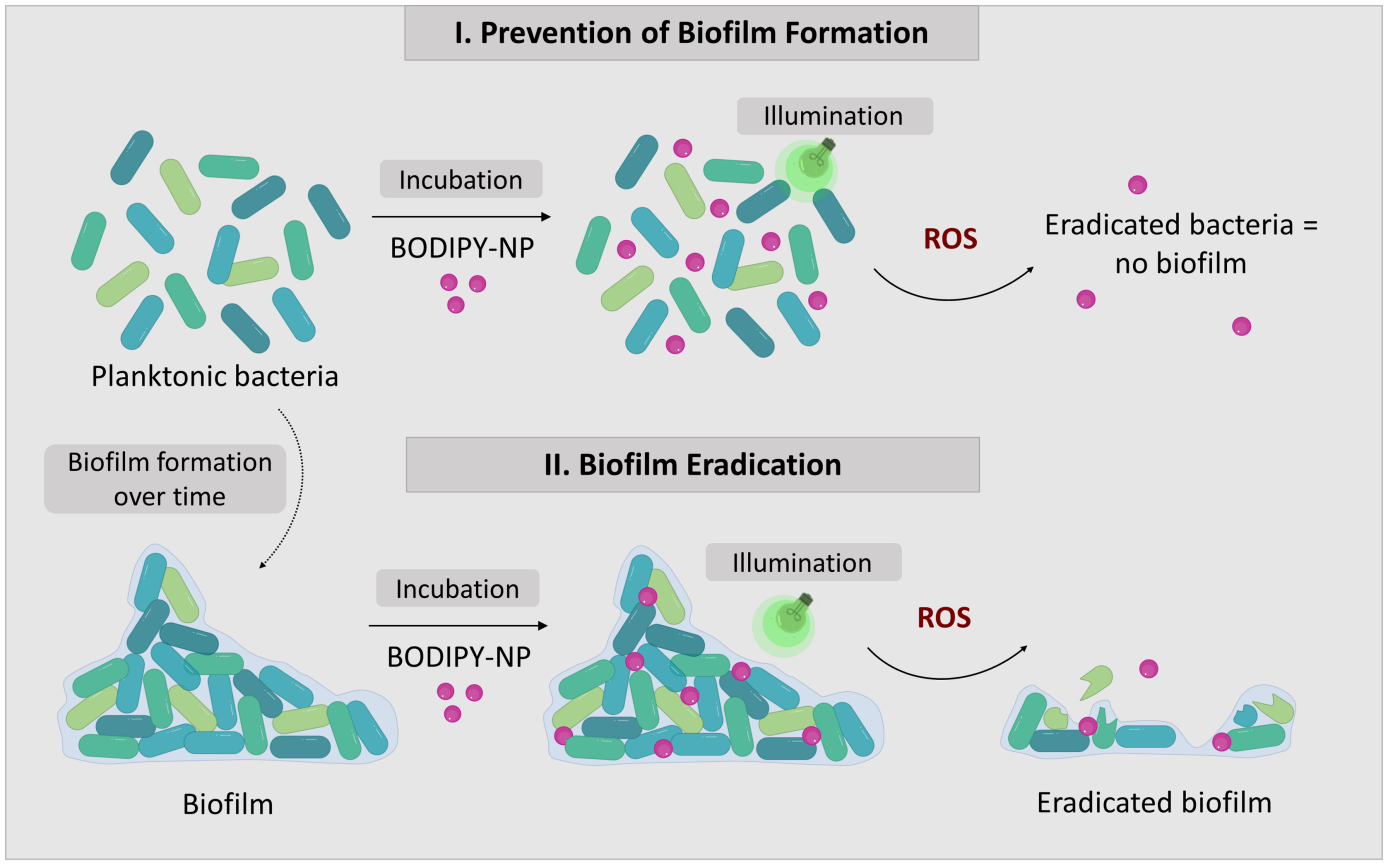
The concept of biofilm eradication utilizing aPDT with BODIPY-loaded NPs. The two approaches to combat biofilms: I. Prevention of biofilm formation by eradication of planktonic bacteria; II. Eradication of existing biofilms.

BODIPY dyes are highly versatile, can be easily prepared and chemically modified and are well suited as PS for aPDT (Ziessel et al., 2007; Bañuelos, 2016). Not only do they exhibit very high photostability, but they also have remarkable photophysical properties, such as their high absorption coefficients and high quantum yields for ROS generation, especially when halogens are introduced to the chromophore core (Gorbe et al., 2019). Here, a diiodinated BODIPY derivative was used due to its highly effective ROS generation, in comparison, to, e.g., the commonly used PS rose bengal (Radunz et al., 2020). Furthermore, BODIPY dyes enable dual use as PS for aPDT and as reporter for fluorescence imaging. One major limitation of BODIPYs is their often very poor water solubility. Thus, the direct application of BODIPYs in aqueous media in relevant concentrations is not feasible. The use of cosolvents (e.g., DMSO) would theoretically be possible, but relatively high concentrations are needed to dissolve adequate amounts of BODIPY. Thus, the risk of the cosolvent influencing the biological system under investigation is high (Summer et al., 2022). Here, the hydrophobic BODIPY dye was embedded into polystyrene NPs that are readily suspendable in aqueous media due to their hydrophilic surface functionalization. The NPs are commercially available, cost effective and have a wide range of applications especially for nano-sized applications such as biosensors or as self-assembling nanostructures (Velev and Kaler, 1999; Kromer et al., 2022). Here, 100 nm polystyrene NPs with a hydrophilic surface functionalization of carboxyl and amine groups were chosen. These NPs are biocompatible, stable in cell culture media and reportedly non-toxic to bacterial cells (Miyazaki et al., 2013; Loos et al., 2014). Especially in the concentration range required for use as a nanocarrier for PS, no negative effects have been reported on the viability or physiology of biofilms (Supplementary Figure S1) (Kromer et al., 2022). The main advantage for the application of these NPs as nanocarriers is their previously reported ability to accumulate well in biofilms (Kromer et al., 2022). The rational design of our approach includes the following steps. First the PS dye was embedded in 100 nm NPs by a previously established dye loading protocol using a swelling method (Behnke et al., 2011). Upon illumination with visible light (530 nm) ROS are generated (Figure 1). For eradication of biofilms the BODIPY-loaded NPs are incubated and accumulated in the biofilm. Photosensitization and ROS generation inside the biofilm leads to the destruction of the bacteria and in case of high effectiveness ultimately the biofilm. The NPs can be applied for the eradication of planktonic cells and subsequently the prevention of biofilm formation or for the eradication of existing biofilms (Figure 2).

### 3.2. Characterization of the NPs and ROS generation

**Figure 3:**
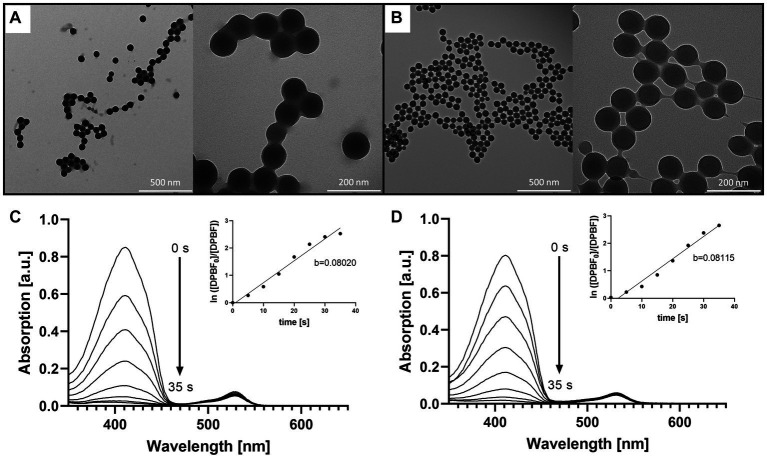
Comparison of the precursor NPs, the free BODIPY dye and the BODIPY-loaded NPs. TEM images of **(A)** the precursor NPs and **(B)** the NPs after incorporation of the BODIPY dye. **(C)** Determination of singlet oxygen generation of the free BODIPY dye. Absorption spectra of DPBF showing decomposition by singlet oxygen generated by free BODIPY dye upon stepwise (7 × 5 s) illumination with a 530 nm LED array. Inset: illumination time-dependent changes in absorbance of DPBF derived from the absorption spectra. **(D)** Determination of singlet oxygen generation of the BODIPY-loaded NPs with the same setup as in **(C)**. Inset: illumination time-dependent changes in absorbance of DPBF derived from the absorption spectra in presence of BODIPY-loaded NPs.

Since the size of the NPs is of major importance influencing the capability of the particles to accumulate in the biofilm, the size of the NPs was determined before and after loading the PS. A change in size could be associated with agglomeration of the particles, which in turn could decrease the stability of the suspension and, subsequently, could affect the accumulation of the NPs in the biofilm. Hence, the dye loading would have had to be limited if these parameters had changed due to the introduction of the dye. The particle size determined by DLS and TEM showed that the size the BODIPY-loaded NPs was not altered (Table 1). Furthermore, TEM images revealed, that the particle shape was also not altered by the incorporation of the dye and remained spherical (Figures 3A,B). The PDI assessment revealed that both the precursor NPs and the dye-containing NPs show a very narrow and comparable size distribution and there was no indication of particle agglomeration. Consequently, the introduction of the BODIPY dye did not affect the particle size, shape, and agglomeration behavior of the NPs. The zeta potential of the precursor NPs and the BODIPY-loaded NPs was determined to − 30.7 ± 1.1 mV and − 22.1 ± 0.6 mV, respectively. It is favorable that the zeta potential is still in the negative range after the incorporation of the dye, since a negative zeta potential is considered biocompatible for polystyrene NPs (Miyazaki et al., 2013; Frankel et al., 2020). Moreover, the diffusion rate of negatively charged nanoparticles is higher than that of positively charged nanoparticles, since the latter are retained via electrostatic interactions (Blanco-Cabra et al., 2022). The diffusion rate is relevant for the accumulation of the particles in the biofilm.

**Table 1:** Particle size and zeta potential of the precursor NPs and dye-loaded NPs.

	Size (TEM) [nm]	Size (DLS) [nm]	PDI	Zeta potential [mV]	Dye loading
NPs	96 ± 12	130 ± 1	0.008 ± 0.006	− 30.7 ± 1.1	–
NPs + BODIPY	90 ± 9	137 ± 2	0.111 ± 0.018	− 22.1 ± 0.6	61.3 ± 5.1 nmol/mg NPs

The dye loading of the particles was determined by dissolving the BODIPY-loaded NPs in THF and photometrical analysis, comparing the BODIPY absorbance with the free dye at 530 nm (Supplementary Figure S2). The dye loading was determined to be 61.3 ± 5.1 nmol per mg NPs (Table 1). No leaking of BODIPY dye from the NPs was observed after 24 h and 72 h in cell culture medium, here M9 minimal medium. This is critical with respect to long-term stability and applicability of the NPs for aPDT. The main factors governing undesired dye leaking are the hydrophilicity of the dye and its solubility in the solvent or matrix surrounding the NPs (Behnke et al., 2011). The absorption and emission spectra of the BODIPY dye were not significantly changed by incorporation into the NPs (Supplementary Figure S3). It was investigated whether embedding the BODIPY dye in the NPs affects or limits the dyes ability to generate ROS. In the case of a compromised ROS generation due to the incorporation of BODIPY dye into the NPs, the effectiveness of the aPDT would be lowered. It would also pose the question if embedding BODIPYs in NPs is a suitable way of application for photodynamic applications or even aPDT. Therefore, DPBF was used as detection molecule in a singlet oxygen assay. In the presence of singlet oxygen, DPBF degrades to a colorless product, thus, the decrease of DPBFs absorption at 410 nm can be used to determine the rate of singlet oxygen generation of the sensitizer (Figures 3C,D). The determined singlet oxygen generation rates prove a similar DPBF degradation of 0.08020/s and 0.08115/s for the free dye and the dye embedded in the NPs, respectively (Figures 3C,D insets). Accordingly, embedding BODIPY into NPs in the concentration tested does not alter the ability of the PS to produce singlet oxygen. It can be assumed that the good oxygen and light transmission of the NPs contributes to the fact that ROS generation is not impaired, even though the dye is loaded in high local concentrations inside the particle. Additionally, effects such as degradation of the dye and inner filter effect seem to be negligible at this concentration.

### 3.3. Eradication of planktonic bacteria and prevention of biofilm formation

The eradication of planktonic bacteria can be an important step in preventing biofilm formation. When planktonic bacteria are present in aqueous media, they can form biofilms. This is not only very problematic in medical environments, but also in water or food processing facilities (Zwirzitz et al., 2020). Especially when affected areas cannot be cleaned with conventional disinfection methods, e.g., due to alcohol-sensitive materials or limited accessibility. Therefore, the performance of BODIPY-loaded NPs was tested against planktonic bacterial cultures and the subsequent biofilm formation was evaluated. Three bacterial species were selected that are known to frequently form pathological biofilms and are of high relevance in the medical field, namely *E. coli, S. aureus,* and *S. mutans. E. coli* is a major cause of urinary tract infections, and its biofilms often lead to chronification of the infection (Eberly et al., 2017). *S. aureus* is a common infector of medical equipment such as catheters or endotracheal tubes (Machado et al., 2012; Zheng et al., 2018) and of surgical sites and burn wounds (Plumet et al., 2022). Contamination of endotracheal tubes often leads to ventilator-associated pneumonia, which most often prolongs the hospital stay in the ICU and has a mortality rate of up to a 20–30%. *S. aureus* and its biofilms are feared in hospitals and intensive care units, especially because of its high pathogenicity and tendency to become multidrug resistant. *S. mutans* is omnipresent in the human oral cavity, but its ability to form biofilms that create an acidic milieu contributes to its high pathogenicity. *S. mutans* is the main cause of dental caries.

**Figure 4:**
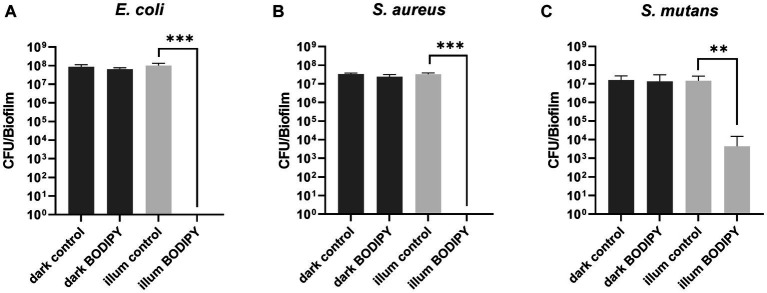
Prevention of biofilm formation after aPDT of planktonic bacteria and determination of subsequent biofilm formation after 24 h. **(A)** Planktonic *E. coli*, **(B)** *S. aureus*, and **(C)** *S. mutans* after 30 min of illumination with a 530 nm LED array. CFU, colony forming units; illum, illuminated. Three independent replicates were performed for each sample. Error bars indicate the standard error of the mean of three independent experiments performed in triplicates. *, **, and *** represent the significant difference to the control determined with an unpaired *t* test with *p* < 0.05, *p* < 0.01, and *p* < 0.001, respectively.

The effectiveness of aPDT with BODIPY-loaded NPs against planktonic bacterial cultures and the subsequent biofilm formation was evaluated for the three bacterial test strains. For this purpose, exponentially growing planktonic bacterial cultures were treated with the BODIPY-loaded NPs and illuminated for 30 min with an LED array at 530 nm. The advantages of this LED array are the easy handling (96 well plates can be mounted on top) and the low susceptibility to errors generated by, e.g., intensity fluctuations. The illumination intensity can be adjusted on the device and ensures good comparability. Unlike lasers, which are often used and are defocused with a lens, the LED array does not need to be readjusted each time. Biofilm formation was observed after 24 h, and the resulting biomass of the biofilm was determined (Figure 4). For this purpose, the biofilms were washed to remove planktonic cells, resuspended in PBS, serially diluted and seeded on agar plates to determine the CFU. For the dark controls, the biofilms were grown for 24 h without NPs and without illumination. When either only the light (illum control) or only the BODIPY-loaded NPs (dark BODIPY) are used, no eradication effect was observed. Only when both are used in combination (illum BODIPY), a strong eradication effect is achieved, leading to the prevention of biofilm formation, even after 24 h post incubation. The eradication of the planktonic cultures results in 100% prevention of biofilm formation for *E. coli* and *S. aureus* and > 99.9% for *S. mutans.* This demonstrates, that aPDT with the BODIPY-loaded NPs is a highly effective method for the treatment of planktonic bacterial cultures and a suitable method for the prevention of biofilm formation.

### 3.4. Biofilm eradication

Although eradicating planktonic bacteria and thus preventing biofilm formation is a valid option, there must also be systems that can combat existing biofilms. Once biofilms have formed and matured, they pose a much greater threat, as they are then very difficult to remove (Koo and Yamada, 2016). One reason for this is that the EPS matrix protects the biofilm by providing structural integrity, thereby increasing its resistance to external influences such as disinfection. It also forms a reservoir for nutrients, hence only a few bacteria need to survive for a biofilm to regrow quickly.

**Figure 5:**
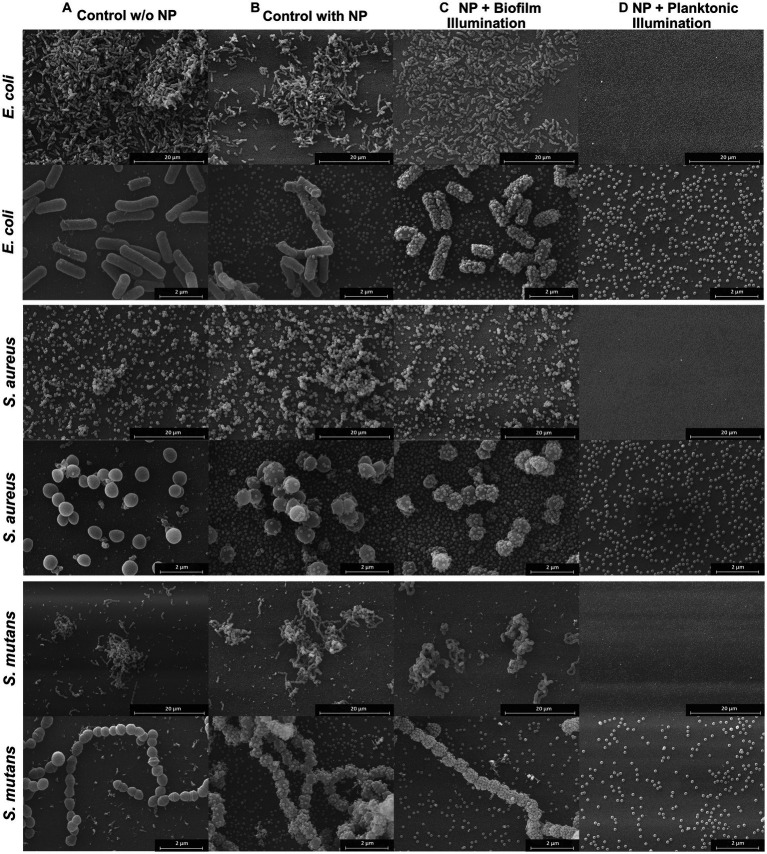
SEM images of *E. coli*, *S. aureus*, and *S. mutans* biofilms treated with aPDT. The four columns display the different treatments from left to right: biofilm control without NPs, biofilm control with NPs and without illumination, biofilm with NPs and illumination, biofilm grown for 24 h after treatment of planktonic culture with NPs and illumination. Additional SEM images can be found in Supplementary Figures S5–S7.

To investigate whether the BODIPY-loaded NPs are able to eradicate biofilms, treatment against *E. coli*, *S. aureus*, and *S. mutans* biofilms was tested and their effect determined. Additionally, planktonic cultures were treated and the subsequent biofilm formation was examined. Each bacterial strain was cultivated separately on glass for 24 h, then incubated with the dye-loaded NPs and illuminated with an LED array at 530 nm. Afterwards, the biofilms were fixed and examined by SEM to investigate the effects of aPDT treatment on the bacteria and biofilm structure (Figure 5). Furthermore, planktonic bacterial cultures were treated as described before (Figure 5D). Controls without NPs and controls with NPs but without illumination were included (Figures 5A,B). Due to the sample preparation for SEM imaging, which includes numerous washing steps, and the loose and fluffy structure of the *S. aureus* and *S. mutans* biofilms, a large proportion of the biofilms were detached from the slides. Therefore, the SEM results can only be used for a comparison between treatment and controls, but not as an indicator for the natural morphology of the biofilms. For all three biofilm test strains, the biofilm structure was not disrupted or altered by the presence of the NPs without illumination compared to the control without nanoparticles. Therefore, dark toxicity of the NPs is not expected. As treatment groups, both biofilms and planktonic cultures were exposed to the NPs and then illuminated. In the treated planktonic samples (Figure 5D), no bacteria or biofilm structures were found in any of the samples. This indicates 100% eradication of planktonic bacteria, as biofilm formation was completely prevented. The results are consistent with the previously obtained results from the biofilm CFU assays and thus demonstrate a good performance of the aPDT NPs in preventing biofilm formation.

In the treated biofilm samples, the number of bacteria and the biofilm structures appear to be reduced (Figure 5C). A determination whether the bacteria present are alive or dead by SEM is very difficult. Although it is normal for dead cells to remain structurally intact during fixation and thus are visible by SEM imaging, the morphology of the cell and the surface structure of the dead cells can be altered (Cheng et al., 2011). The cells can become deformed and wrinkled, indicating that the intracellular content had leaked out (García-Salinas et al., 2018). Studies have also found, that dead cells emptied of their cellular content can still have almost intact cell walls (Diogo et al., 2017). In this case, however, the structural change of the membrane is difficult to observe due to the membrane of the bacteria being completely covered with NPs after the treatment. This is a very unusual observation revealing a significant change in comparison to the NPs controls. The attachment of NPs to the bacterial membranes is eminent in all three bacterial species.

**Figure 6:**
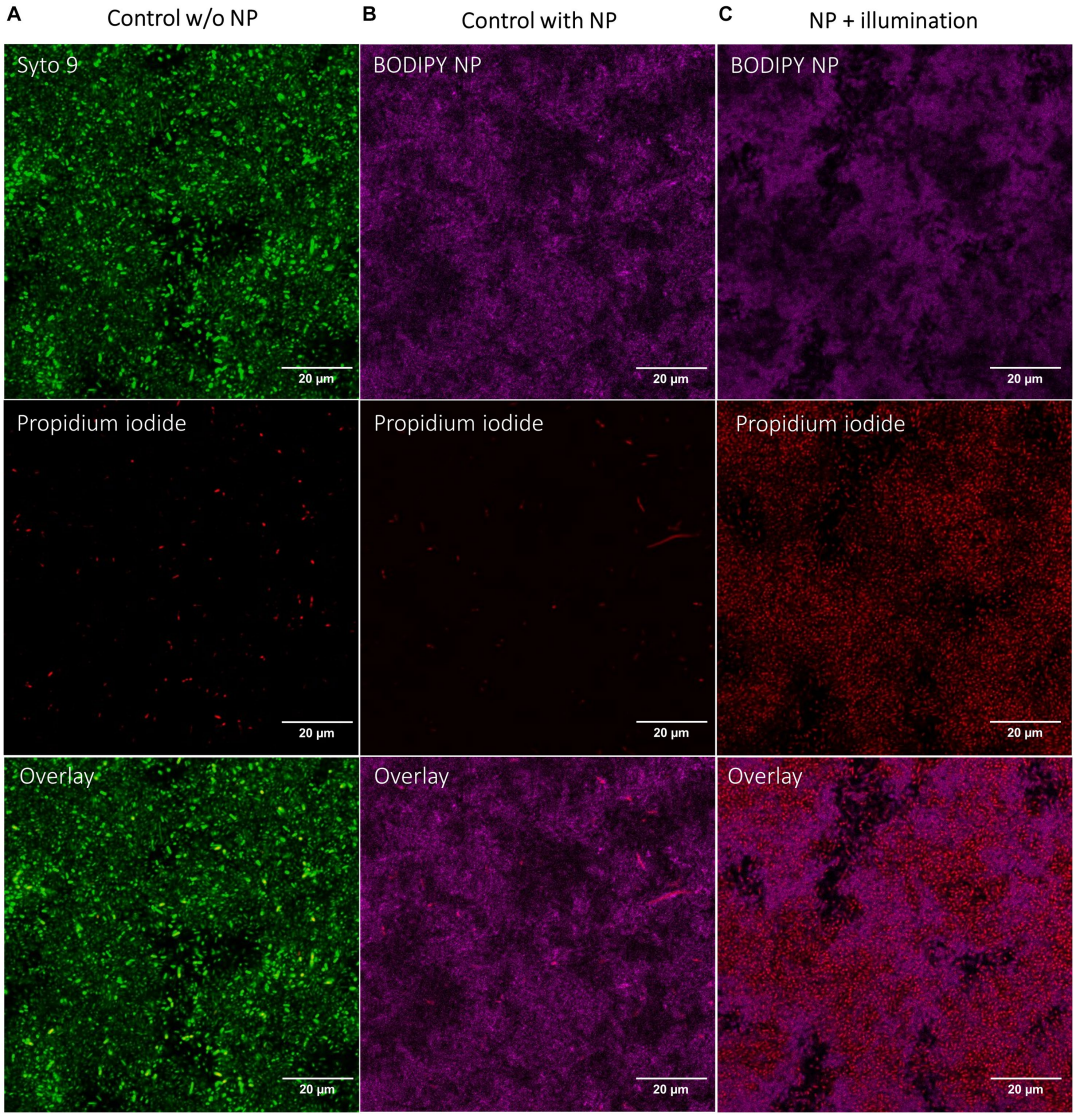
CLSM images of *E. coli* biofilms after aPDT with BODIPY-loaded NPs. **(A)** Control biofilm without NPs with illumination, **(B)** Biofilm incubated with NPs without illumination, **(C)** Biofilm incubated with NPs and with illumination. BODIPY-loaded NPs (magenta), Live/Dead stain of bacteria with propidium iodide for dead cells (red) and Syto9 for live cells (green). Scale bar is 20 μm.

Since it was not possible to distinguish the live from the dead bacteria with SEM, the dead cells were visualized by staining with propidium iodide and investigation with CLSM after aPDT treatment (Figure 6). In an untreated control, the living cells were additionally stained with Syto9. A Syto9 stain is not possible in the BODIPY-loaded NPs treated samples due to an overlap of the emission of BODIPY and Syto9. The NPs accumulated in the biofilm can be imaged by the fluorescence of BODIPY. The control biofilm shows a large number of live cells (green signal) and a small number of dead cells (red signal) (Figure 6A). The NPs control (Figure 6B) incubated only with BODIPY-loaded NPs but not illuminated shows few dead cells, comparable to the control without NPs. When the biofilm and the NPs accumulated within are illuminated, a very high number of dead cells is observed (Figure 6C). This indicates that the bacteria and the biofilm structure itself are still intact, but the cells are dead after the aPDT treatment. This is consistent with the SEM images where bacterial structures were also visible after treatment, but most likely dead.

**Figure 7:**
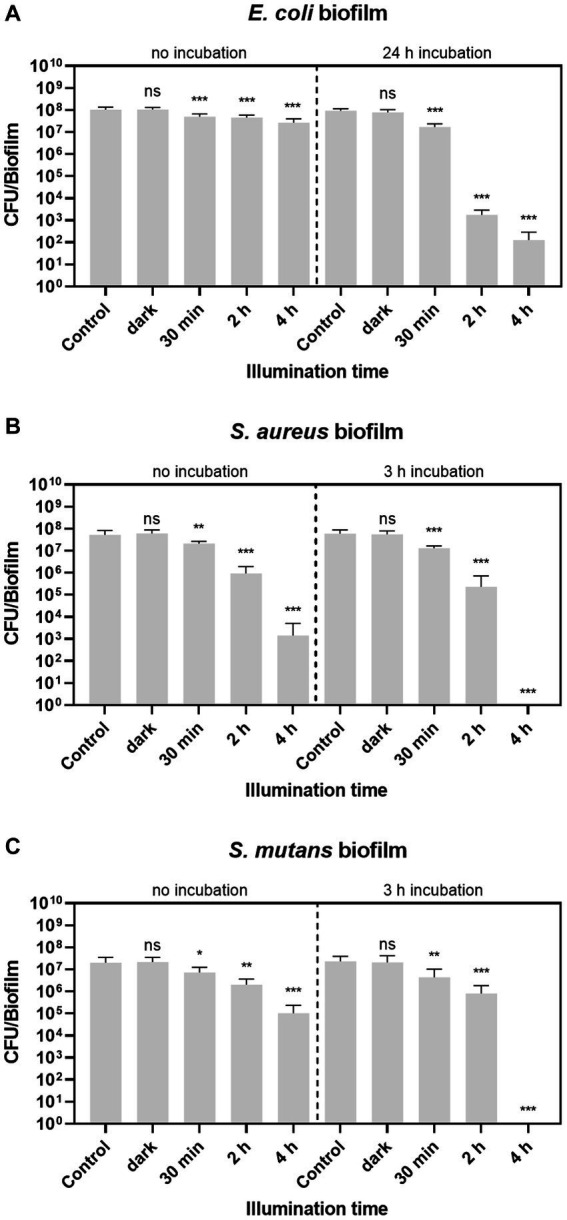
Biofilm eradication with BODIPY-loaded NPs. **(A)** *E. coli*, **(B)** *S. aureus*, and **(C)** *S. mutans* biofilms were illuminated either with or without previous incubation of the dye-loaded NPs. The CFU per biofilm were determined after different illumination times (30 min, 2 h and 4 h), biofilm samples without NPs after 30 min and 4 h (control), and biofilms exposed to NPs but without illumination after 30 min and 4 h (dark). Error bars indicate the standard error of the mean of three independent experiments performed in triplicates. *, **, and *** represent the significant difference to the control determined with an unpaired *t* test with *p* < 0.05, *p* < 0.01, and *p* < 0.001, respectively.

The microscopic images revealed that treatment of biofilms with aPDT leads to a high number of dead cells in the biofilm. Treatment of planktonic cultures with aPDT can even prevent biofilm formation altogether. To determine the effectiveness of biofilm eradication by aPDT treatment with BODIPY-loaded NPs, the number of viable bacteria in the biofilms after treatment was quantified. For this purpose, biofilms of three bacterial species were grown in 96-well plates for 24 h. After a washing step with PBS, the BODIPY-loaded NPs were added to the biofilms, incubated for a given time period and were illuminated with the LED array. To determine the viable fraction of the biofilms after aPDT treatment, the biofilms were then washed, resuspended in PBS, serially diluted and the resulting CFU determined (Figure 7). The empty 96-well plates were then stained with crystal violet to ensure a complete removal of biofilm from the plate. Previous studies have found a clear correlation between illumination intensity and eradication efficiency, with higher intensity leading to greater bacterial eradication (Shrestha et al., 2014; Gillespie et al., 2017; Sun et al., 2019). Considering the goal of our study to achieve the highest possible eradication efficiency, we opted for the highest illumination intensity of the LED array. Initially, the biofilms were illuminated for 30 min as with the planktonic cultures. However, although an illumination time of 30 min resulted in effective eradication for the planktonic cultures, this was not the case for the much more robust biofilms. Here, only ~0.3–0.4 log units of bacteria in the biofilms were eradicated after 30 min of illumination. Increased illumination times of 2 h and 4 h resulted in significantly better eradication of the biofilms. After 4 h, the aPDT treatment was able to eradicate 0.65 log units of *E. coli*, 5.8 log units of *S. aureus* and 3.3 log units of *S. mutans* compared to the control.

Since a successful eradication method aims for 100% eradication, the setup was further optimized. For this purpose, the NPs were first incubated in the biofilm and then irradiated. The optimal incubation time was determined using CLSM by determining the accumulation of BODIPY-loaded NPs into the biofilm at different time points. The time point up to which an increase of particles in the biofilm could be observed was determined as the needed incubation time. It was observed that the time required for the NPs to accumulate in the biofilm varies depending on the biofilm test strain, therefore different incubation times were chosen. Incubation times of 3 h were sufficient for *S. aureus* and *S. mutans* biofilms. For the *E. coli* biofilms, a significantly longer incubation time of 24 h was required to achieve sufficient accumulation in the biofilm. Representative images of optimizing the incubation time of *E. coli* biofilms are shown in Supplementary Figure S4.

For all three biofilms tested, the eradication effect increased with increasing illumination time and is generally higher when the NPs were previously incubated into the biofilm. No dark toxicity of the BODIPY-loaded NPs is observed in the dark controls for any of the three bacterial biofilm types. This confirms that the eradication effect is exclusively achieved by aPDT. Remarkably, after 4 h of treatment, two of the three biofilm species, namely *S. aureus* and *S. mutans*, were eradicated by 100%. *E. coli* biofilms were eradicated by 6.65 log units corresponding to 99.99986%.

## 4. Discussion

This study has demonstrated that nanoparticles for aPDT can be a powerful tool for the eradication of planktonic bacteria and bacterial biofilms. It was shown that the aPDT eradication effect increased with increasing illumination time and was generally higher when the NPs were previously incubated into the biofilm. The efficiency of the BODIPY-loaded NPs is thus higher when ROS generation takes place in close proximity to the bacteria inside the biofilm and not in the medium. Since the generated ROS have a short lifespan and thus a short diffusion distance, the spatial proximity to the bacteria is a very important factor for the effectiveness. Additionally, it was found that the incubation times needed for a sufficient accumulation in the biofilm vary depending on the biofilm species. The NPs penetration into and movement within the biofilm is considered to be driven primarily by diffusion (Ikuma et al., 2015). The most important factors influencing the diffusion of NPs into the biofilm are particle properties such as size, charge and hydrophobicity (Hollmann et al., 2021). However, the nature of the biofilm also plays a very important role. Here, the pore size and hydrophobicity of the biofilm are particularly important, but also the charge and chemical gradient of the biofilm and its matrix (Peulen T. O. and Wilkinson, 2011; Sahle-Demessie and Tadesse, 2011). Since the same NPs were used here for all three biofilms, the structure and nature of the biofilms is most likely the main factor for the kinetic of NPs penetration in the biofilms. *E. coli* forms very dense biofilms with quite small extracellular spaces and with smaller pore size. Hence, longer incubation times were needed for *E. coli* biofilms than for *S. aureus* and *S. mutans.* These biofilms tend to have a looser structure with more extracellular matrix and larger pore size (Hou et al., 2019). This finding aligns with the previously stated hypotheses that the pore size of a biofilm significantly influences penetration of NPs into that biofilm.

A main feature of the aPDT method described here are the very high eradication rates of up to 100%. Biofilms have the ability to quickly rebuild large populations, even if only a few specimens survive a disinfection treatment. Not eliminating all bacteria in a biofilm is a major shortcoming of most published studies utilizing PS-loaded NPs (Klepac-Ceraj et al., 2011; Usacheva et al., 2016; Anju et al., 2018, 2019). Therefore, a key feature of a successful eradication method is to achieve 100% eradication to permanently eliminate biofilms. In this study, 100% eradication was achieved for, *S. aureus* and *S. mutans.* In the case of *E. coli* biofilms, a very small fraction of the biofilm survives the treatment regardless. Since the rate of surviving bacteria in *E. coli* biofilms decreases steadily with the illumination time, it is reasonable to assume that 100% eradication can be achieved with longer illumination times for *E. coli* as well. In general, the difference in the susceptibility of gram-negative and gram-positive bacteria to aPDT can be attributed to the different cell wall structure of the bacteria (Malik et al., 1992; Bekmukhametova et al., 2020). Gram-negative bacteria, in contrast to gram-positive bacteria, have a more complex cell wall structure due to an additional lipopolysaccharide containing membrane as the outermost layer. This membrane provides additional protection against ROS, as it is not enough to disturb the outer-membrane structure alone, but the cytoplasmic membrane must be disrupted as well (Malik et al., 1992). Gram-negative bacteria and biofilms can be just as harmful as gram-positive bacteria. Therefore, aPDT agents that are able to successfully eliminate gram-negative bacteria need to be further developed and optimized.

These studies also include SEM images that revealed that the NPs attach to the bacterial membranes after the illumination. This observation was eminent in all three bacterial species. The lack of membrane potential, as it occurs in dead cells, could be a reason for the adhesion of the NPs to the cells. Furthermore, changes in the cell membrane could be involved as well. The outer cell membranes of viable bacteria are typically negatively charged due to the presence of molecules such as lipopolysaccharides and carboxylate substituents on their surface (Wilson et al., 2001). Thus, the NPs, which are also negatively charged, have no electrostatic attraction to the bacteria. However, when the bacteria are damaged by aPDT leading to cell wall ruptures, substances can leak from inside the bacteria. The release of intracellular components might result in the exposure of positively charged molecules or ions on the bacterial surface, causing a shift in the overall charge from negative to neutral or positive (Kłodzińska et al., 2010; Ferreyra Maillard et al., 2021). This might lead to the binding of NPs to the bacterial surfaces by electrostatic attraction, as observed by SEM. It is therefore reasonable to conclude that bacteria with a large number of NPs adhering to their surface are dead.

In summary, the design, preparation, characterization, and application of a BODIPY-loaded NPs tool for aPDT of bacteria and their corresponding biofilms was described. The BODIPY-NPs proved to be highly effective for the prevention of biofilm formation as well as the eradication of biofilms. With its simple preparation and easy application, the aPDT system stands in contrast to existing methods, which most often require a complex manufacturing and application and therefore lack practical suitability. It has the potential to be used as a seminal and universal disinfection agent for the much-needed treatment of pathogenic bacteria and biofilms. Furthermore, the multitargeted mechanism of action of aPDT leads to a demonstrably lower development of bacterial resistance (Vatansever et al., 2013; Bekmukhametova et al., 2020). By combining the NPs with substances that enable active targeting, such as lectins or antibodies, aPDT can potentially be applied more selective. Thus, in a mixed bacterial population, only the pathogenic bacteria could be eradicated, allowing potential applications where bacteria are beneficial such as the skin or gut microbiome. The BODIPY-loaded NPs could also be used for theranostic applications, where not only eradication but also diagnosis of the bacteria via imaging techniques (here by fluorescence) is given. It is also possible to combine the PS with a bactericidal drug in one particle to achieve a dual mode of action, thus further increasing the efficiency.

## Data availability statement

The original contributions presented in the study are included in the article/Supplementary material, further inquiries can be directed to the corresponding author/s.

## Author contributions

CK: Conceptualization, Data curation, Formal Analysis, Investigation, Visualization, Writing – original draft. KS: Supervision, Writing – original draft. SR: Investigation, Writing – review & editing. DT: Investigation, Writing – review & editing. PL: Writing – review & editing. AL: Supervision, Writing – review & editing. HT: Conceptualization, Supervision, Writing–original–draft.

## Figures and Tables

**Table:** 

EPS	extracellular polymeric substances
ROS	Reactive oxygen species
PDT	Photodynamic therapy
PS	Photosensitizer
aPDT	Antimicrobial photodynamic therapy
BODIPY	Boron-dipyrromethene
NPs	Nanoparticles
THF	Tetrahydrofuran
PDI	Polydispersity index
DLS	Dynamic light scattering
TEM	Transmission electron microscope
DPBF	1, 3-diphenylisobenzofuran
LB	Luria–Bertani
CFU	Colony forming units
CLSM	Confocal laser scanning microscopy
SEM	Scanning electron microscopy
