# Supplementary material for: ROS generating BODIPY loaded nanoparticles for photodynamic eradication of biofilms
Source: Front Microbiol. 2023 Oct 12;14:1274715. doi: 10.3389/fmicb.2023.1274715 (PMC10615615; doi:10.3389/fmicb.2023.1274715)
Supplement: Supplementary file 1 [file Data_Sheet_1.docx]

Supplementary Material

**
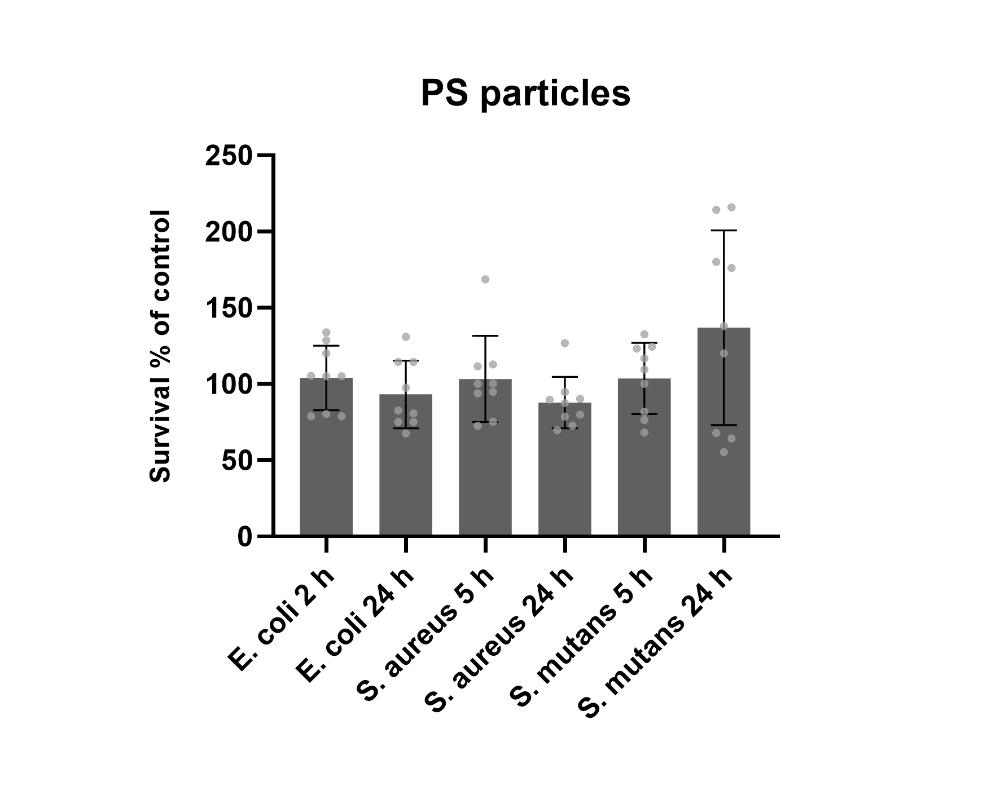
**

**Supplementary Figure 1.** Viability after incubation of PS NP without dye. Survival % of control of *E. coli*, *S. aureus* and *S. mutans* biofilms after various incubation times. All experiments were carried out as three independent replicates. None of the tested incubation times showed a significant toxic effect. Thus, within the testing conditions and experimental setup the PS NP are biocompatible and have no effect on the bacteria.


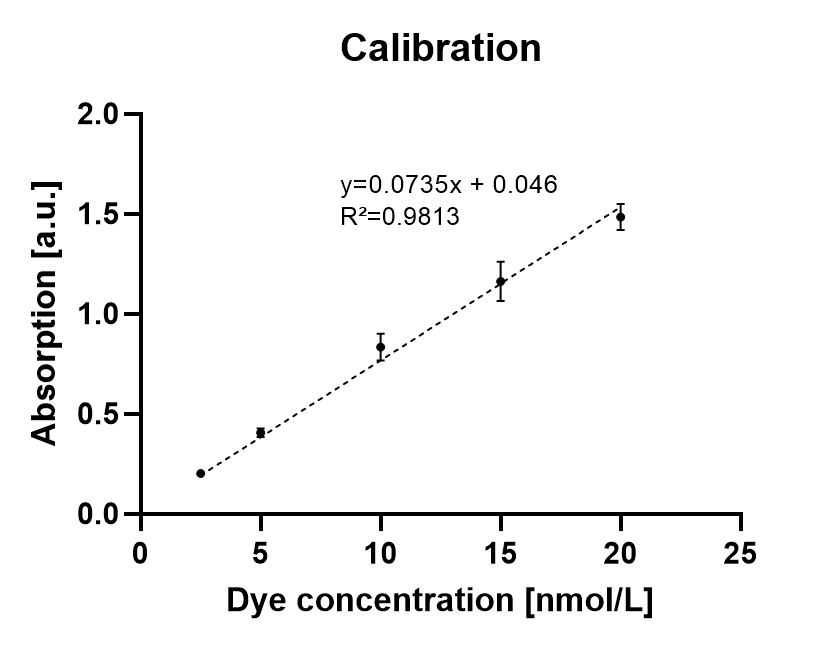


**Supplementary Figure 2.** Calibration curve for determination of BODIPY dye loading of the BODIPY-loaded NPs.

$$E\lambda={log}_{10} \frac{\mathrm{Io}}{I} = \varepsilon\lambda\cdot c \cdot d$$

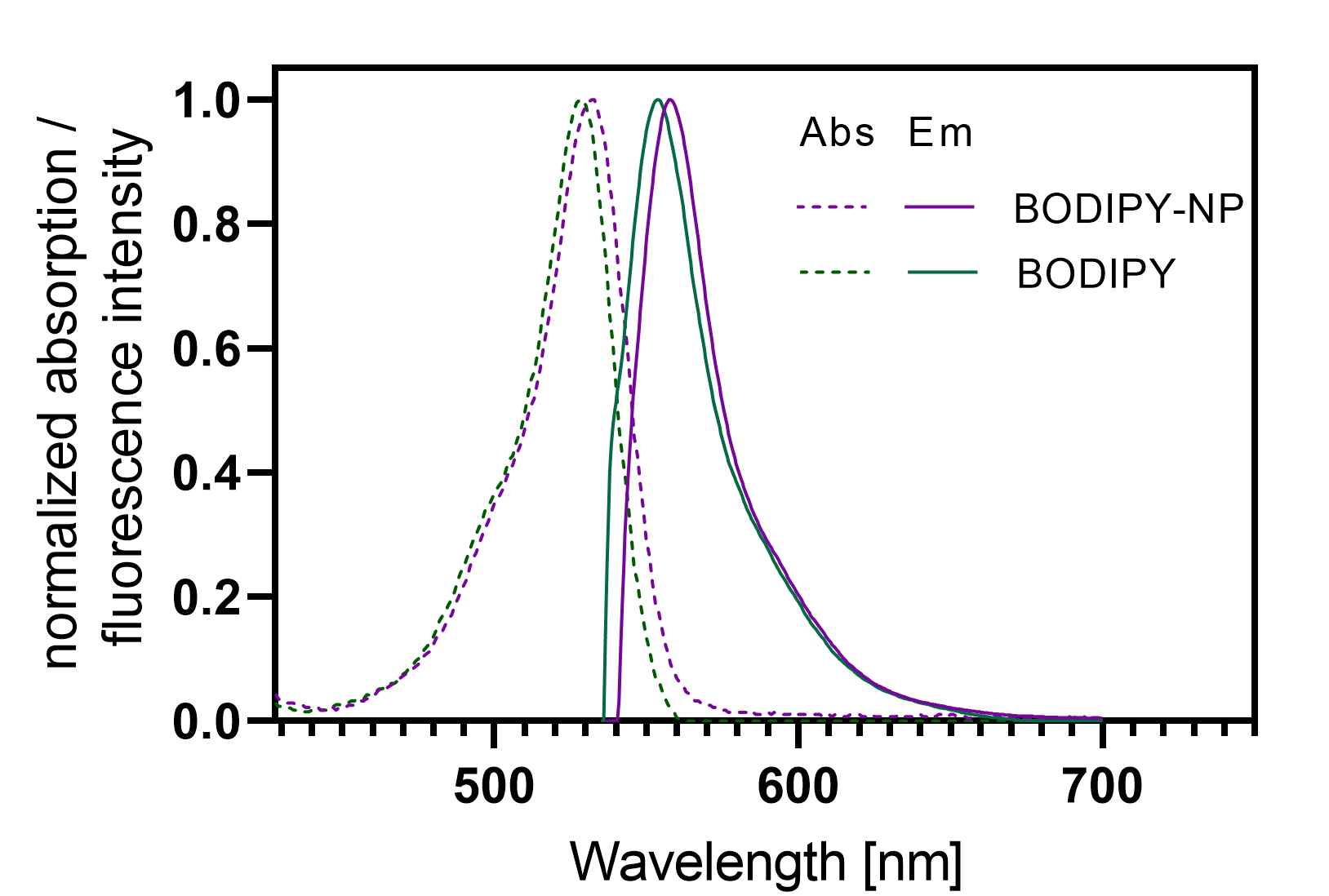


**Supplementary Figure 3.** Absorption and emission spectra of the free BODIPY dye and the BODIPY-loaded NPs in 50% ACN. Excitation for the Emission spectra was 530 nm.


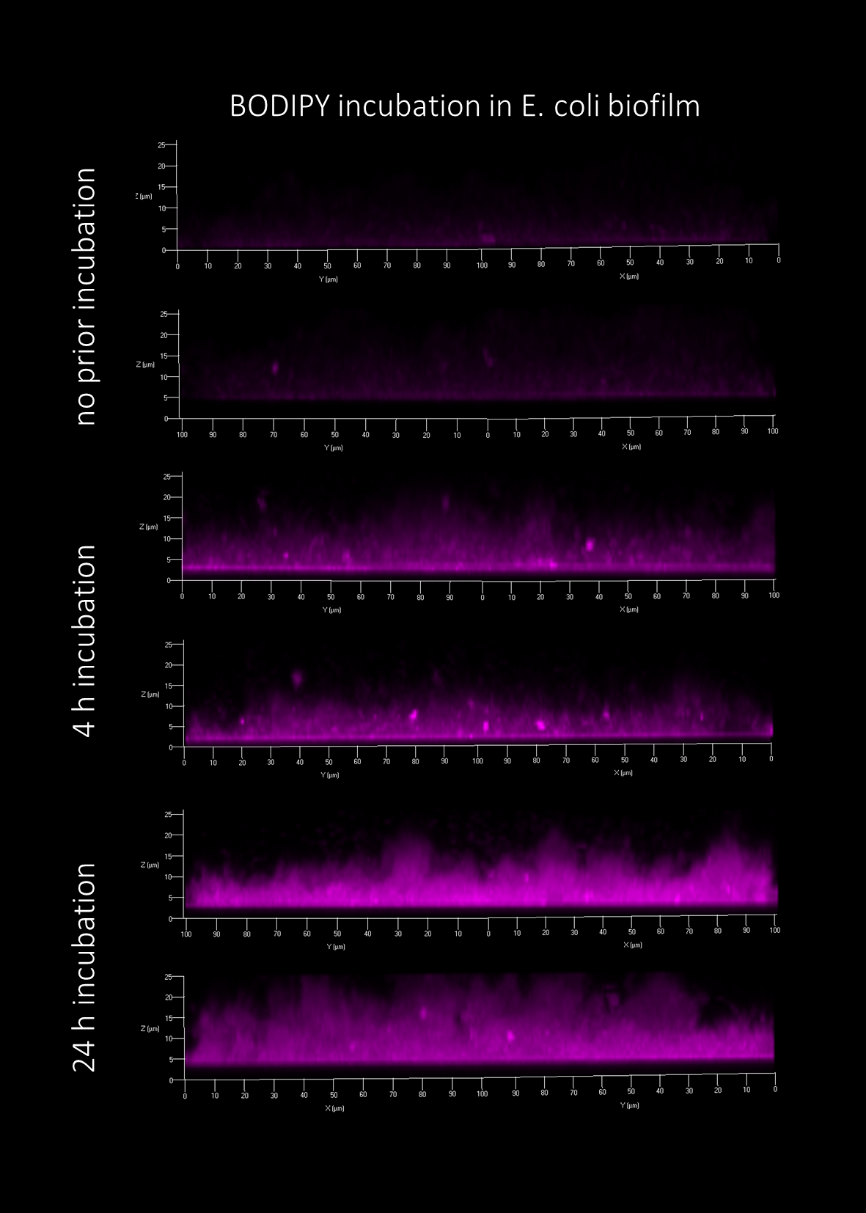


**Supplementary Figure 4.** CLSM images of the incubation time optimization for *E. coli* biofilms. BODIPY dye (magenta) was imaged in *E. coli* biofilms with no incubation, 4 hours incubation and 24 hours incubation. The goal was a sufficient penetration into the biofilm and accumulation in the biofilm.


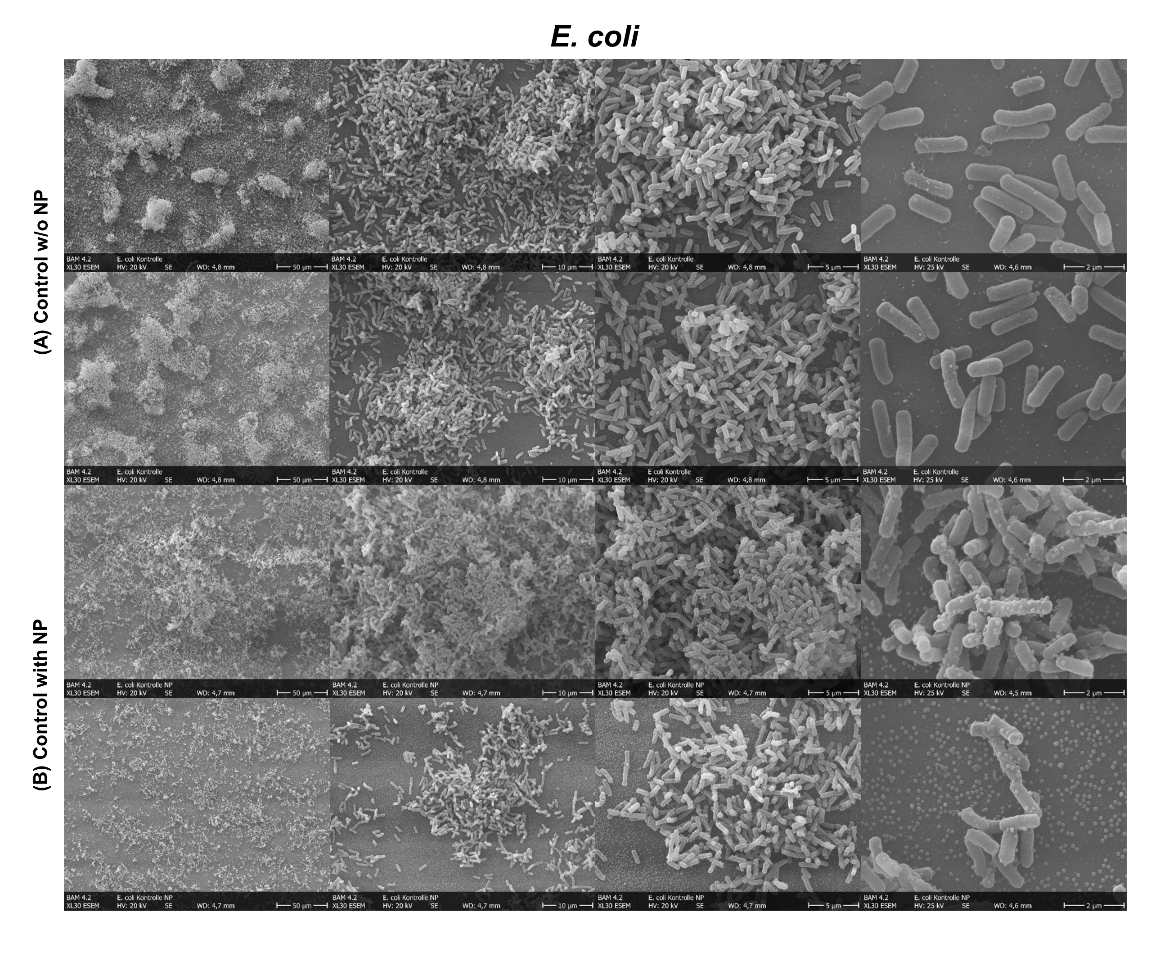

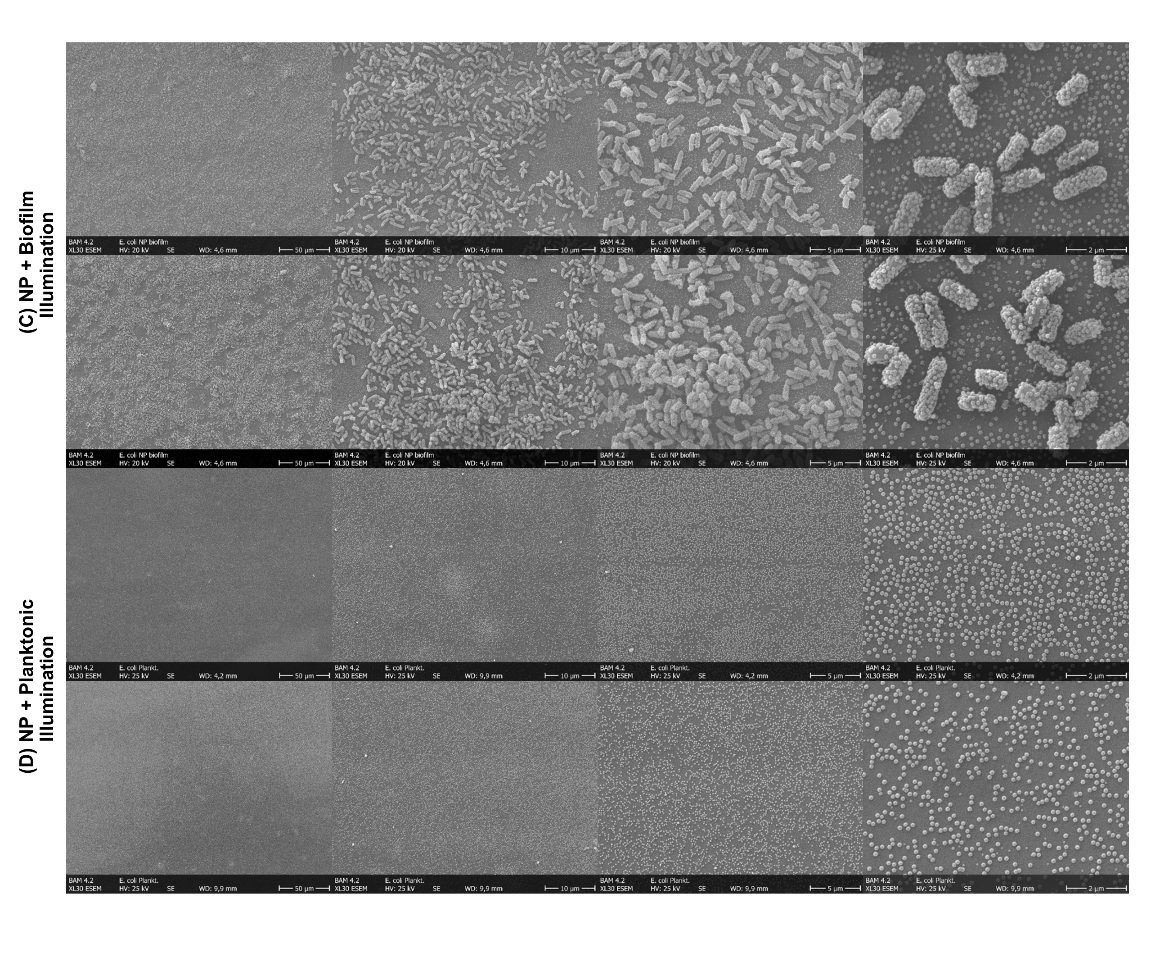


**Supplementary Figure 5.** SEM images of *E. coli* biofilms treated with aPDT. The four columns display increasing magnifications from left to right. a) Biofilm control without NP, b) biofilm control with NP and without illumination, c) biofilm with NP and illumination, d) biofilm grown for 24 hours after treatment of planktonic culture with NP and illumination.


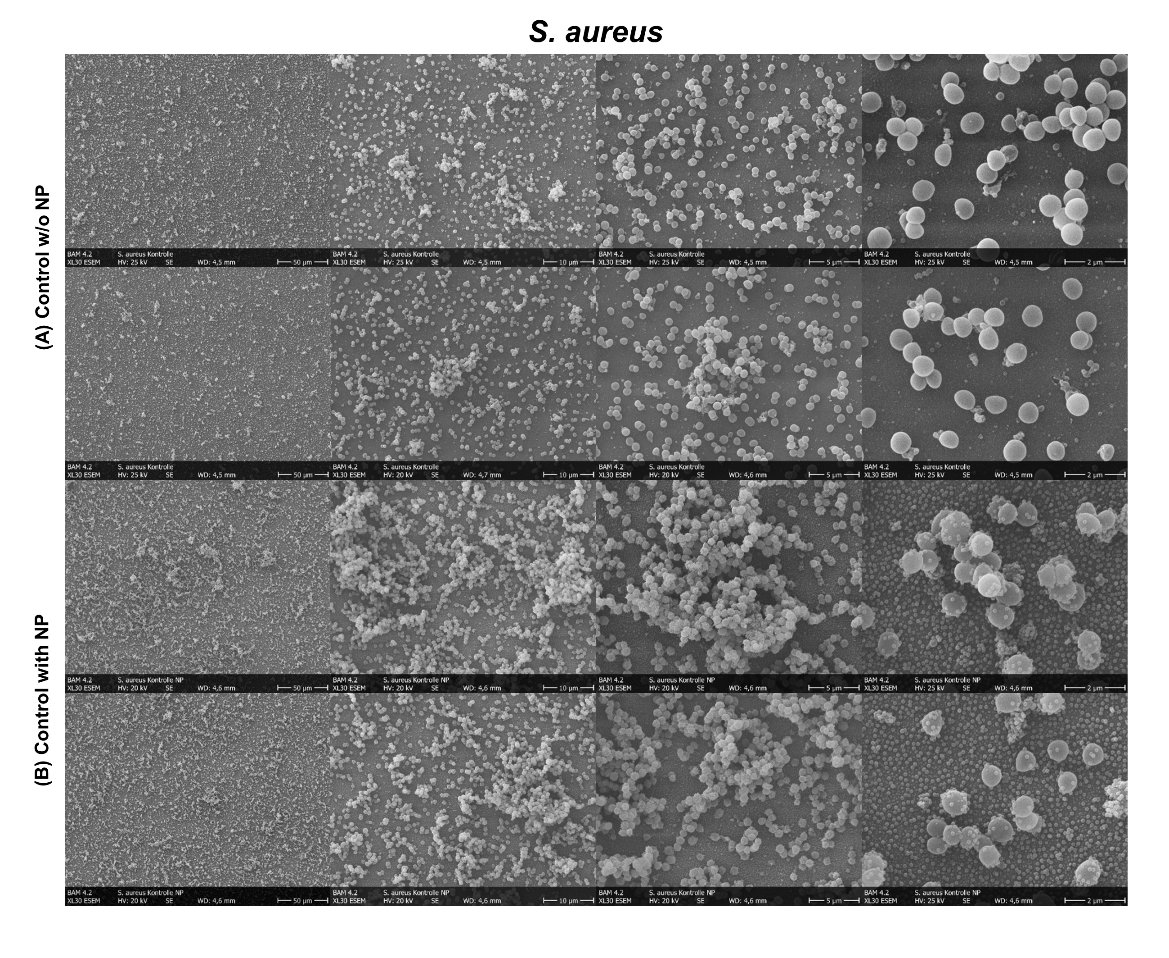

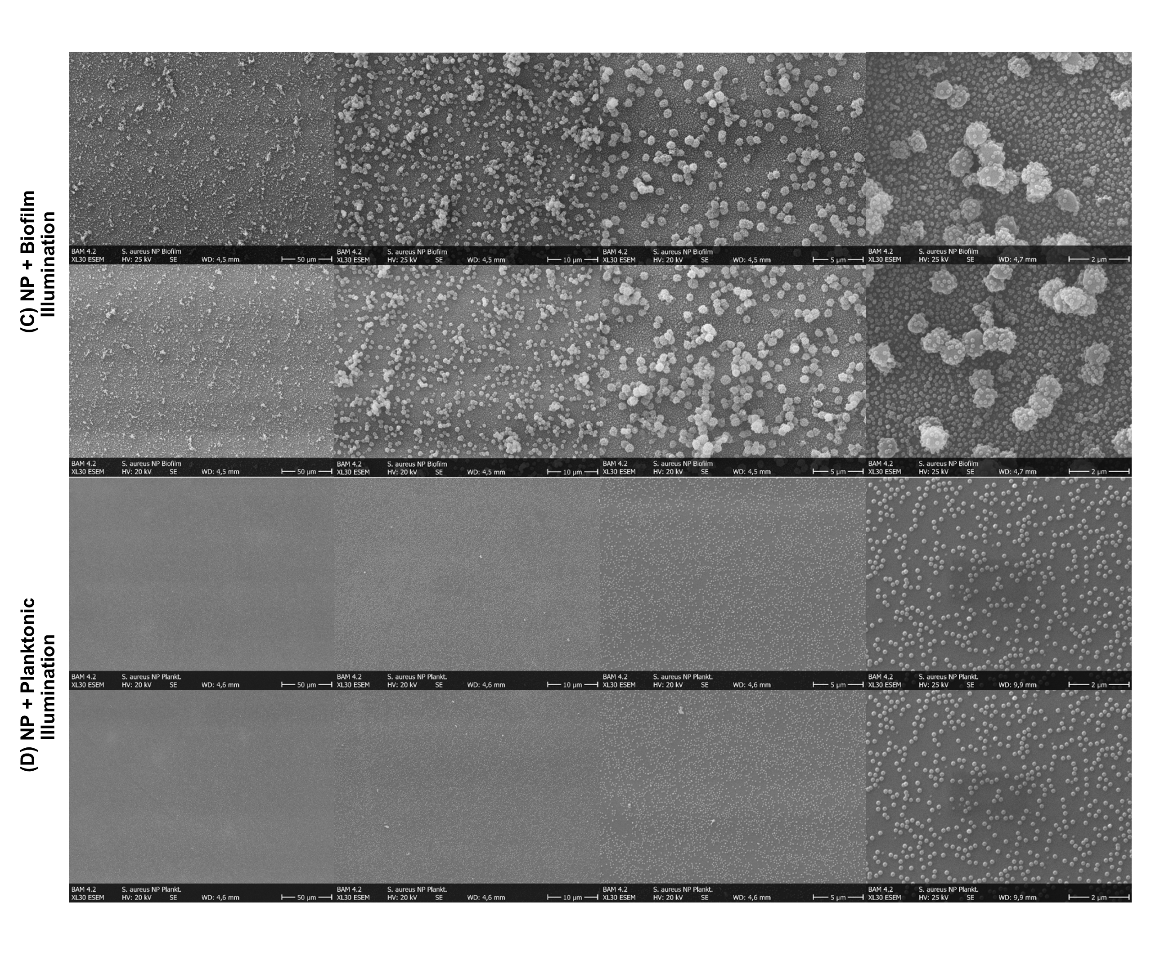


**Supplementary Figure 6.** SEM images of *S. aureus* biofilms treated with aPDT. The four columns display increasing magnifications from left to right. a) Biofilm control without NP, b) biofilm control with NP and without illumination, c) biofilm with NP and illumination, d) biofilm grown for 24 hours after treatment of planktonic culture with NP and illumination.


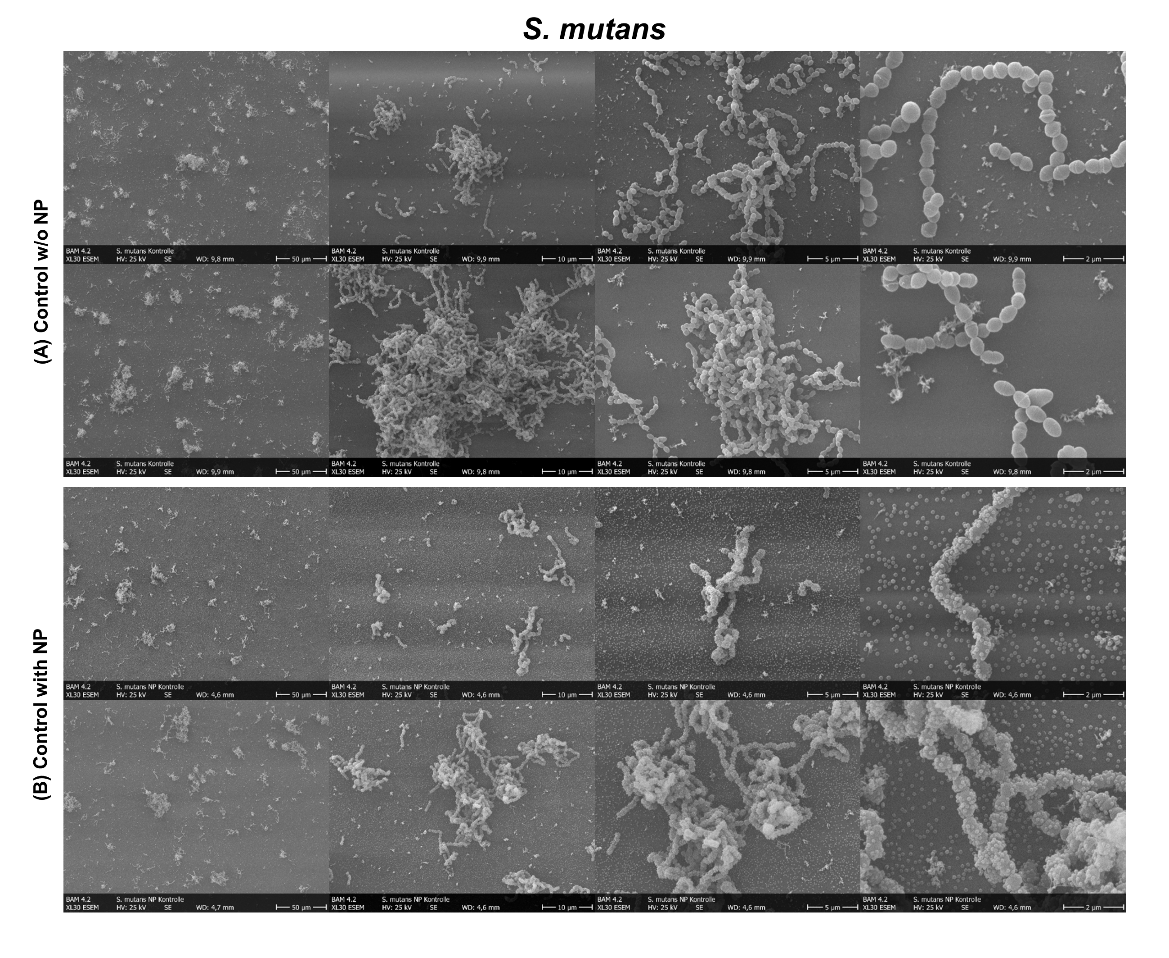

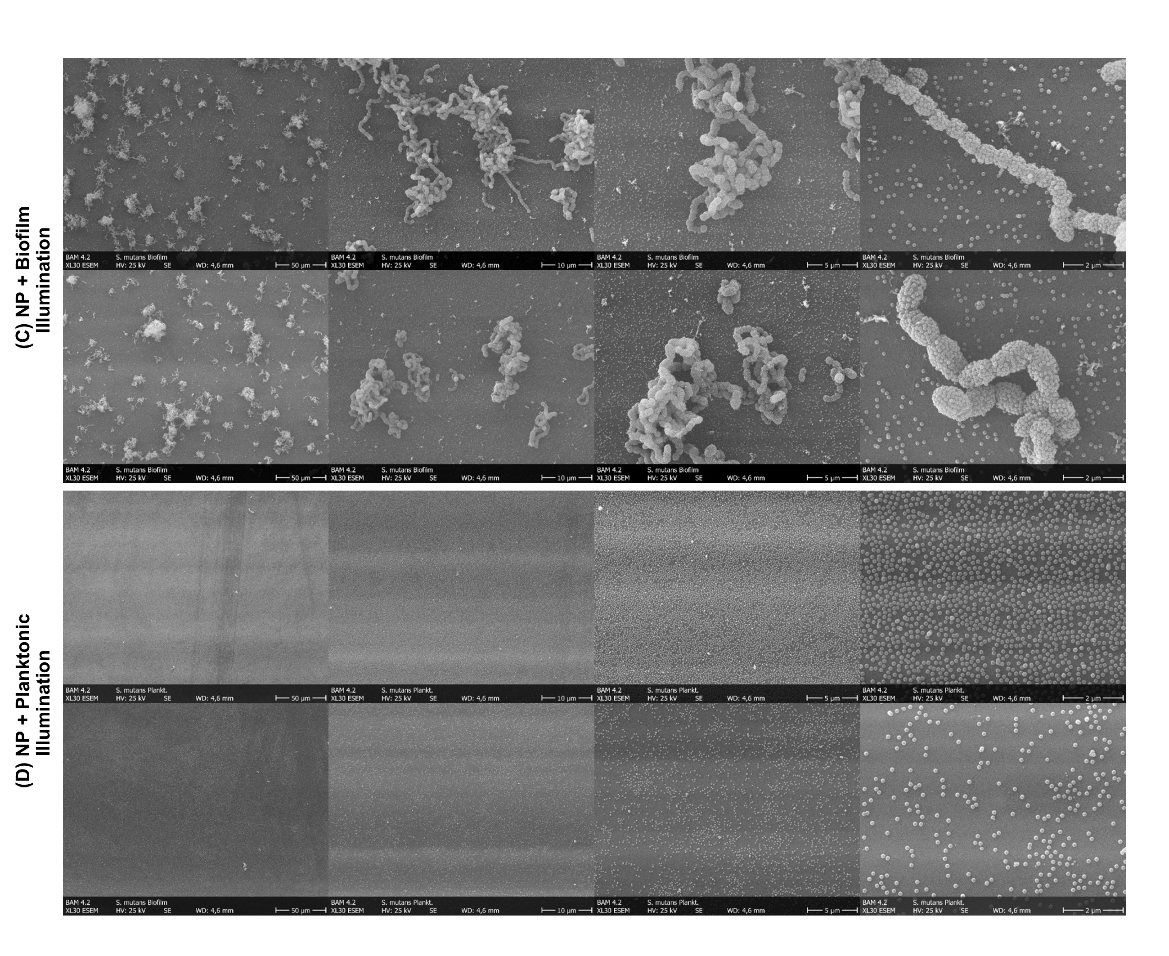


**Supplementary Figure 7.** SEM images of *S. mutans* biofilms treated with aPDT. The four columns display increasing magnifications from left to right. a) Biofilm control without NP, b) biofilm control with NP and without illumination, c) biofilm with NP and illumination, d) biofilm grown for 24 hours after treatment of planktonic culture with NP and illumination.
